# Estimated prevalence of multiple chronic conditions throughout adulthood using data from the *All of Us* Research Program

**DOI:** 10.1101/2024.10.17.24315661

**Published:** 2024-10-18

**Authors:** Xintong Li, Caitlin Dreisbach, Carolina M. Gustafson, Komal Patel Murali, Theresa A. Koleck

**Affiliations:** 1 Goergen Institute for Data Science, University of Rochester, Rochester, NY, USA; 2 School of Nursing, University of Rochester, Rochester, NY, USA; 3 School of Nursing, University of Pittsburgh, Pittsburgh, PA, USA; 4 Rory Meyers College of Nursing, New York University, New York, NY, USA

**Keywords:** Electronic health records, Multiple chronic conditions, Prevalence, Secondary data analysis

## Abstract

Estimation of multiple chronic condition (MCC) prevalence throughout adulthood provides a critical reflection of MCC burden. We analyzed electronic health record codes for 58 conditions to estimate MCC prevalence for *All of Us* (*AoU*) Research Program adult participants (N=242,828). Approximately 76% of *AoU* participants were diagnosed with MCCs, with over 40% having 6 or more conditions and prevalence increasing with age; the most frequently occurring MCC combinations varied by age category (i.e., mental health conditions in early adulthood and physical health conditions in middle adulthood through advanced old age). We report notable prevalence of MCC throughout adulthood and variability in MCC condition combinations by age category in *AoU* participants. These findings highlight the need for targeted, innovative care modalities and population health initiatives to address MCC burden throughout adulthood.

## Introduction

Multiple chronic conditions (MCCs) is defined as the presence of two or more chronic conditions such as Alzheimer’s disease, arthritis, asthma, cancer, chronic kidney disease, diabetes, heart failure, and hypertension (1). Approximately 80% of adults 65 years of age and older in the United States (U.S.) are living with MCCs (2). Diagnosis with MCCs, however, is not limited to older adults. One study estimated that 22% of young adults, 18–34 years of age, are diagnosed with MCCs (3). Moreover, research suggests that more contemporary generations of adults have greater MCC burden and are diagnosed with MCCs at earlier ages than previous generations (4). Estimation of the prevalence of MCCs throughout all stages of adulthood is a critical reflection of MCC burden; it is important to examine the prevalence of MCCs broadly using regularly updated data sources to inform targeted prevention and management strategies as well as resource prioritization. Thus, the purpose of this study is to estimate the prevalence of MCCs for adult participants in the National Institutes of Health *All of Us* (*AoU*) Research Program dataset – a curated disease and population-agnostic, longitudinal biomedical dataset of diverse individuals living in the U.S. – to gain a deeper understanding of MCCs throughout adulthood (i.e., early adulthood, middle adulthood, late-middle adulthood, late adulthood, and advanced old age).

## Materials and Methods

We conducted a cross-sectional analysis using the *AoU* Controlled Tier version 7 data release (date of last access 16/06/2024) in the secure, cloud-based *AoU* Researcher Workbench platform. This study was exempt from human subjects’ approval as only deidentified data were analyzed. Using the Cohort Builder tool, we identified all adult participants 18 years of age or older who consented to share electronic health record (EHR) data and had at least one International Classification of Diseases-10th Revision (ICD-10) code documented to ensure the availability of EHR data (N=242,828). For all participants meeting these eligibility criteria, we extracted relevant ICD-10 codes and the corresponding date of documentation for 58 chronic conditions (Table 1) cataloged in the Centers for Medicare & Medicaid Services (CMS) Chronic Conditions Data Warehouse (5). Due to reliance on ICD-10 codes for both cohort identification and as the source of condition information, we limited participant visits to on or after October 1, 2015, i.e., initiation of ICD-10 implementation. Thus, data included in this analysis are from October 1, 2015, to July 1, 2022, i.e., latest date available in the version 7 release.

Then, we used the Python programming language in Jupyter Notebook to chronologically order the chronic condition ICD-10 codes by date of documentation for each participant. By distinguishing the onset date of the second unique chronic condition, we were able to identify participants with MCCs. We calculated the number of participants with 0, 1, 2, 3, 4, 5, and 6+ chronic conditions in the overall cohort and stratified by age category, i.e., early adulthood (18–39 years), middle adulthood (40–49 years), late-middle adulthood (50–64 years), late adulthood (65–74 years), and advanced old age (75–89 years) (6,7). We used the age at first documentation of the second chronic condition for categorization. Finally, we calculated the frequency of unique MCC condition combinations by chronic condition count (i.e., 2, 3, 4, 5, and 6+) and age category.

## Results

Approximately 76% (n=183,753) of participants were diagnosed with MCCs, with over 40% of participants (n=98,885) diagnosed with 6 or more conditions (Table 2). Of the remaining participants, 11% (n=27,122) were diagnosed with a single chronic condition, and 13% (n=31,953) did not have a chronic condition documented. Overall, participants (Table 3) were on average 49.8 (SD=16.7) years of age and primarily women (60.9%), White (54.6%), not Hispanic or Latino (77.3%), married/partnered (49.9%), highly educated (68.7% with some college or higher), insured (92.2%), and not currently employed (47.7%). Compared to participants diagnosed with 0 or 1 conditions, participants diagnosed with MCC were older (mean age of 52.8 years vs. 40.4 years). In addition, a higher percentage of participants with MCCs were men (37.4% vs. 33.8%), White (58.6% vs. 49.6%), not Hispanic/Latino (79.1% vs. 71.4%), insured (93.1% vs. 89.5%), ever married (75.7% vs. 66.4%), and not currently employed (52.3% vs. 33.5%), with a much greater frequency of retired participants (26.4% vs. 10.2%), compared to participants diagnosed with 0 or 1 conditions. The percentage of participants within an age category diagnosed with 6 or more chronic conditions increases with age (Table 2), from 33.78% in early adulthood to 68.04% in advanced old age.

The top five most frequently diagnosed MCC condition combinations in each age category are detailed in Table 4. The most frequently occurring combination in each age categorization was as follows: early adulthood – anxiety disorders and depression, bipolar, or other depressive mood disorders (n=845); middle and late middle adulthood – fibromyalgia, chronic pain and fatigue and rheumatoid arthritis/osteoarthritis (n=204 and n=457, respectively); and late adulthood and advanced old age – hyperlipidemia and hypertension (n=200 and n=59, respectively).

## Discussion

We estimated MCC prevalence in adults living in the U.S. from early adulthood to advanced old age using ICD-10 codes from participants enrolled in *AoU*. We found that approximately 76% of participants have MCC, with higher prevalence as age increases. These findings emphasize the value of *AoU* in identification of MCC burden throughout adulthood. We believe the percentage of adults with MCCs in this study is higher than previous reports due to our robust inclusion of 58 chronic conditions, selected based on the CMS Chronic Conditions Data Warehouse. The characteristics of adult participants who are enrolled in *AoU* and consented to share EHR data – i.e., women, White, not Hispanic/Latino, married/partnered, highly educated, insured, and not currently employed – must also be considered when interpreting the reported estimates and generalizing results to the broader U.S., and worldwide, population.

While sampling bias should be taken into account, this analysis contributes to our understanding of the impact of MCCs. Given the notable prevalence of MCCs across adulthood, there is a continued need for innovative care modalities and public health initiatives to address the multifaceted nature of MCC (8). MCC are associated with clinical complexity characterized by increased symptom burden, high health care utilization and cost, and extensive caregiver strain (9). Ongoing research efforts that seek to both reduce the burden of MCCs on the healthcare system and the development of person- and family-centered care strategies across the healthcare continuum are needed (9).

We found that the most frequent MCC condition combinations vary by age category. Notably, a combination of mental, rather than physical, health conditions (i.e., anxiety disorders and depression, bipolar, or other depressive mood disorders) was the most common MCC combination in early adulthood. Following early adulthood, combinations of physical health conditions were the most common (e.g., hyperlipidemia and hypertension in late adulthood and advanced old age). For precision health, characterizing MCC profiles and the most frequently co-occurring conditions in different age categories can inform targeted prevention and treatment approaches and allow clinicians to better anticipate and manage comorbidities in their patients (10).

In summary, this brief report fills an important knowledge gap and provides a foundation for future studies on MCC correlates, predictors, and outcomes by communicating current MCC prevalence estimates, that capture a wider range of chronic conditions, across the adult lifespan.

## Figures and Tables

**Table 1.** Chronic conditions included in the multiple chronic condition prevalence analysis (N=58)

Acute myocardial infarction	HIV/AIDS
ADHD, conduct disorder, and hyperkinetic syndrome	Hyperlipidemia
Alzheimer’s disease	Hypertension
Anemia^1^	Hypothyroidism
Anxiety disorders^2^	Intellectual disabilities and related conditions
Asthma	Ischemic heart disease
Atrial fibrillation and flutter	Learning disabilities and developmental delays^3^
Autism spectrum disorders	Leukemias and lymphomas
Benign prostatic hyperplasia	Liver disease, cirrhosis and other liver conditions
Blindness and visual impairment	Migraine and chronic headache
Cancer, breast	Mobility impairments
Cancer, colorectal	Multiple sclerosis and transverse myelitis
Cancer, endometrial	Muscular dystrophy
Cancer, lung	Non-Alzheimer’s dementia
Cancer, prostate	Obesity
Cancer, urologic	Osteoporosis
Cataract	Parkinson’s disease and secondary parkinsonism
Cerebral palsy	Peripheral vascular disease
Chronic kidney disease	Personality disorders
Chronic obstructive pulmonary disease	Pneumonia
Cystic fibrosis and other metabolic developmental disorders	Pressure and chronic ulcers
Deafness and hearing impairment	Rheumatoid arthritis/osteoarthritis
Depression, bipolar, or other depressive mood disorders	Schizophrenia and other psychotic disorders
Diabetes	Spina bifida and other congenital anomalies of the nervous system
Epilepsy	Spinal cord injury
Fibromyalgia, chronic pain and fatigue	Stroke/transient ischemic attack
Glaucoma	Substance use disorders^4^
Heart failure and non-ischemic heart disease	Traumatic brain injury and nonpsychotic mental disorders due to brain damage
Hip/pelvic fracture	Viral hepatitis

ADHD=Attention-deficit/hyperactivity disorder; HIV/AIDS=Human immunodeficiency virus/Acquired immunodeficiency syndrome.

International Classification of Disease-10 Revision (ICD-10) codes from the Centers for Medicare and Medicaid Services Chronic Condition Warehouse were used identify diagnosis of a chronic condition.^5^

1 Includes ICD-10 codes for “anemia” and “sickle cell anemia”

2 Includes ICD-10 codes for “anxiety disorders” and “post-traumatic stress disorder”

3 Includes ICD-10 codes for “learning disabilities” and “other developmental delays”

4 Includes ICD-10 codes for “alcohol use disorders”; “drug use disorders”; opioid use disorder 1, 2, 3 and 4; and “tobacco use disorders”, excluding Z71.41, Z71.42, Z71.51, Z71.52, and Z71.6

**Table 2.** Number of participants with multiple chronic conditions overall and by age categorization

Condition count	n	%
**Overall** (N=183,753)		
2	24,039	9.90
3	21,910	9.02
4	20,075	8.27
5	18,844	7.76
6+	98,885	40.72
**Early adulthood, 18–39 years** (n=40,237)		
2	9,327	23.18
3	7235	17.98
4	5,648	14.04
5	4,435	11.02
6+	13,592	33.78
**Middle adulthood, 40–49 years** (n=28,906)		
2	4,139	14.32
3	3,689	12.76
4	3,244	11.22
5	3,052	10.56
6+	14,782	51.14
**Late middle adulthood, 50–64 years** (n=66,705)		
2	6,786	10.17
3	6,945	10.41
4	6,895	10.34
5	6,715	10.07
6+	39,364	59.01
**Late adulthood, 65–74 years** (n=35,549)		
2	2,778	7.81
3	3,019	8.49
4	3,227	9.08
5	3,462	9.74
6+	23,063	64.88
**Advanced old age, 75–89 years** (n= 11,289)		
2	811	7.18
3	844	7.48
4	919	8.14
5	1,034	9.16
6+	7,681	68.04

**Table 3.** Participant characteristics (N=242,828)

	Overall (N = 242,828)	0 or 1 conditions (n = 59,075)	MCC (n = 183,753)	p-value^1^
	Mean (SD) or Freqency (%)	Mean(SD) or Freqency (%)	Mean(SD) or Freqency (%)	
**Age** (years)^2^				
	49.8 (16.7)	40.4 (16.5)	52.8 (15.7)	<0.001
**Race**				
Asian	6,514 (2.7)	2,816 (4.8)	3,698 (2.0)	<0.001
Black	43,431 (17.9)	9,991 (16.9)	33,440 (18.2)	
Middle Eastern/North African	1,413 (0.6)	462 (0.8)	951 (0.5)	
Native Hawaiian/Pacific Islander	303 (0.1)	78 (0.1)	225 (0.1)	
White	137,039 (56.4)	29,314 (49.6)	107,725 (58.6)	
Multiracial	4,233 (1.7)	1,291 (2.2)	2,942 (1.6)	
Unknown	49,895 (20.5)	15,123 (25.6)	34,772 (18.9)	
**Ethnicity**				
Not Hispanic or Latino	187,586 (77.3)	42,193 (71.4)	145,393 (79.1)	<0.001
Hispanic or Latino	46,011 (18.9)	14,978 (25.4)	31,033 (16.9)	
Unknown/ Not Reported Ethnicity	9,231 (3.8)	1,904 (3.2)	7,327 (4.0)	
**Gender**				
Man	88,732 (36.5)	19,956 (33.8)	68,776 (37.4)	<0.001
Woman	147,991 (60.9)	37,727 (63.9)	110,264 (60.0)	
Non-Binary/Transgender/Other Gender	1,622 (0.7)	479 (0.8)	1,143 (0.6)	
Unknown	4,483 (1.8)	913 (1.5)	3,570 (1.9)	
**Health Insurance Status**				
Insured	223,842 (92.2)	52,857 (89.5)	170,985 (93.1)	<0.001
Uninsured	11,268 (4.6)	4,074 (6.9)	7,194 (3.9)	
Unknown	7,718 (3.2)	2,144 (3.6)	5,574 (3.0)	
**Employment Status**				
Employed	99,435 (40.9)	31,910 (54.0)	67,525 (36.7)	<0.001
Unemployed	20,477 (8.4)	5,634 (9.5)	14,843 (8.1)	
Unable to work	27,250 (11.2)	2,899 (4.9)	24,351 (13.3)	
Retired	54,590 (22.5)	6,025 (10.2)	48,565 (26.4)	
Homemaker	8,917 (3.7)	2,704 (4.6)	6,213 (3.4)	
Student	4,594 (1.9)	2,515 (4.3)	2,079 (1.1)	
Multiple	18,653 (7.7)	4,977 (8.4)	13,676 (7.4)	
Unknown	8,912 (3.7)	2,411 (4.1)	6,501 (3.5)	
**Annual Income**				
Less than 10,000	31,274 (12.9)	7,510 (12.7)	23,764 (12.9)	<0.001
10,000 to 25,000	28,491 (11.7)	5,702 (9.7)	22,789 (12.4)	
25,000 to 35,000	17,271 (7.1)	4,234 (7.2)	13,037 (7.1)	
35,000 to 50,000	19,232 (7.9)	4,769 (8.1)	14,463 (7.9)	
50,000 to 75,000	25,428 (10.5)	6,066 (10.3)	19,362 (10.5)	
75,000 to 100,000	19,563 (8.1)	4,752 (8.0)	14,811 (8.1)	
100,000 to 150,000	23,765 (9.8)	6,221 (10.5)	17,544 (9.5)	
150,000 to 200,000	10,873 (4.5)	3,020 (5.1)	7,853 (4.3)	
Greater than 200,000	15,186 (6.3)	4,331 (7.3)	10,855 (5.9)	
Unknown	51,745 (21.3)	12,470 (21.1)	39,275 (21.4)	
**Highest Level of Education**				
Never Attended	367 (0.2)	58 (0.1)	309 (0.2)	<0.001
Up to High School	7,710 (3.2)	1,671 (2.8)	6,039 (3.3)	
Some High School	14,131 (5.8)	3,351 (5.7)	10,780 (5.9)	
High School Graduate/GED	46,474 (19.1)	11,122 (18.8)	35,352 (19.2)	
Some College	62,658 (25.8)	13,233 (22.4)	49,425 (26.9)	
College Graduate	53,993 (22.2)	15,107 (25.6)	38,886 (21.2)	
Advanced Degree	50,164 (20.7)	12,847 (21.7)	37,317 (20.3)	
Unknown	7,331 (3.0)	1,686 (2.9)	5,645 (3.1)	
**Marital Status**				
Married/Living with Partner	121,282 (49.9)	30,287 (51.3)	90,995 (49.5)	<0.001
Divorced/Separated	42,814 (17.6)	7,351 (12.4)	35,463 (19.3)	
Widowed	14,286 (5.9)	1,626 (2.8)	12,660 (6.9)	
Never Married	56,246 (23.2)	17,710 (30.0)	38,536 (21.0)	
Unknown	8,200 (3.4)	2,101 (3.6)	6,099 (3.3)	

MCC=multiple (i.e., 2 or more) chronic conditions

1 p-values calculated using chi-squared tests.

2 Age of participants on October 1, 2015, i.e., the initiation of International Classification of Diseases-10th Revision (ICD-10) code implementation, for comparison between groups. Please note that age at first documentation of the second chronic condition, not age on October 1, 2015, was used for age categorization for participants diagnosed with MCC.

**Table 4.** Most frequently diagnosed multiple chronic condition combinations in each age category

Condition combinations	n
**Early adulthood, 18–39 years** (n=40,237)	
Anxiety disorders; Depression, bipolar, or other depressive mood disorders	845
Anemia; Obesity	355
Anxiety disorders; Depression, bipolar, or other depressive mood disorders; Substance use disorders	340
Anxiety disorders; Fibromyalgia, chronic pain and fatigue	238
Anxiety disorders; Depression, bipolar, or other depressive mood disorders; Fibromyalgia, chronic pain and fatigue	216
**Middle adulthood, 40–49 years** (n=28,906)	
Fibromyalgia, chronic pain and fatigue; Rheumatoid arthritis/osteoarthritis	204
Anxiety disorders; Depression, bipolar, or other depressive mood disorders	168
Hyperlipidemia; Hypertension	104
Hypertension; Substance use disorders	103
Hypertension; Obesity	84
**Late middle adulthood, 50–64 years** (n=66,705)	
Fibromyalgia, chronic pain and fatigue; Rheumatoid arthritis/osteoarthritis	457
Hyperlipidemia; Hypertension	385
Hypertension; Substance use disorders	216
Diabetes; Hyperlipidemia; Hypertension	204
Diabetes; Hypertension	173
**Late adulthood, 65–74 years** (n=35,549)	
Hyperlipidemia; Hypertension	200
Fibromyalgia, chronic pain and fatigue; Rheumatoid arthritis/osteoarthritis	182
Cataract; Glaucoma	94
Hyperlipidemia; Hypertension; Rheumatoid arthritis/osteoarthritis	85
Hypertension; Rheumatoid arthritis/osteoarthritis	84
**Advanced old age, 75–89 years** (n=11,289)	
Hyperlipidemia; Hypertension	59
Fibromyalgia, chronic pain and fatigue; Rheumatoid arthritis/osteoarthritis	46
Cataract; Glaucoma	44
Hyperlipidemia; Hypertension; Ischemic heart disease	38
Diabetes; Hyperlipidemia; Hypertension	26

## Data Availability

No new data were generated or analyzed in support of this brief report. Researchers interested in registering for access to the All of Us Research Program data can visit: https://www.researchallofus.org/
